# Not performing an OGTT results in underdiagnosis, inadequate risk assessment and probable cost increases of (pre)diabetes in Han Chinese over 40 years: a population-based prospective cohort study

**DOI:** 10.1530/EC-18-0372

**Published:** 2018-12-05

**Authors:** Xiang Hu, Qiao Zhang, Tian-Shu Zeng, Jiao-Yue Zhang, Jie Min, Sheng-Hua Tian, Hantao Huang, Miaomiao Peng, Nan Zhang, Mengjiao Li, Qing Wan, Fei Xiao, Yan Chen, Chaodong Wu, Lu-Lu Chen

**Affiliations:** 1 Department of Endocrinology, Union Hospital, Tongji Medical College, Huazhong University of Science and Technology, Wuhan, China; 2 Department of Cardiovascular Surgery, Union Hospital, Tongji Medical College, Huazhong University of Science and Technology, Wuhan, China; 3 Yiling Hospital, Yichang, China; 4 Institute of Big Data and Internet Innovation, Hunan University of Commerce, Changsha, China; 5 Department of Nutrition and Food Science, Texas A&M University, College Station, Texas, USA

**Keywords:** diabetes, prediabetes, OGTT, detecting strategies, Han Chinese

## Abstract

**Objective:**

To explore the influence by not performing an oral glucose tolerance test (OGTT) in Han Chinese over 40 years.

**Design:**

Overall, 6682 participants were included in the prospective cohort study and were followed up for 3 years.

**Methods:**

Fasting plasma glucose (FPG), 2-h post-load plasma glucose (2h-PG), FPG and 2h-PG (OGTT), and HbA1c testing using World Health Organization (WHO) or American Diabetes Association (ADA) criteria were employed for strategy analysis.

**Results:**

The prevalence of diabetes is 12.4% (95% CI: 11.6–13.3), while the prevalence of prediabetes is 34.1% (95% CI: 32.9–35.3) and 56.5% (95% CI: 55.2–57.8) using WHO and ADA criteria, respectively. 2h-PG determined more diabetes individuals than FPG and HbA1c. The testing cost per true positive case of OGTT is close to FPG and less than 2h-PG or HbA1c. FPG, 2h-PG and HbA1c strategies would increase costs from complications for false-positive (FP) or false-negative (FN) results compared with OGTT. Moreover, the least individuals identified as normal by OGTT at baseline developed (pre)diabetes, and the most prediabetes individuals identified by HbA1c or FPG using ADA criteria developed diabetes.

**Conclusions:**

The prevalence of isolated impaired glucose tolerance and isolated 2-h post-load diabetes were high, and the majority of individuals with (pre)diabetes were undetected in Chinese Han population. Not performing an OGTT results in underdiagnosis, inadequate developing risk assessment and probable cost increases of (pre)diabetes in Han Chinese over 40 years and great consideration should be given to OGTT in detecting (pre)diabetes in this population. Further population-based prospective cohort study of longer-term effects is necessary to investigate the risk assessment and cost of (pre)diabetes.

## Introduction

Diabetes is a silent killer affecting humans, with continuing growth of incidence, prevalence, costs and death (1). It is estimated that in the Chinese adult population, the overall prevalence of diabetes is 11.6% (2) and 50.1% may have had prediabetes, an important risk factor of overt diabetes and cardiovascular disease (2). Diabetes leads to complications that cause profound psychological and physical distress, putting a huge burden on health care systems (3, 4). The availability of safe and effective therapies for diabetes patients reduces morbidity and mortality by preventing or delaying complications (3). Moreover, diabetes development (5) and the rate of diabetes onset can be significantly decreased in prediabetes individuals with particular interventions (6). Therefore, early detection of (pre)diabetes enables prevention of the development of (pre)diabetes, initiation of patient-centered management to improve glycemic control and minimize complications (3).

There is often a long presymptomatic phase before the diagnosis of type 2 diabetes, which is frequently not diagnosed until its complications appear. Undiagnosed (pre)diabetes has even higher risk of developing complications due to no intervention adopted (3). Approximately one-fourth of the US population may have undiagnosed diabetes (6), while most people with type 2 diabetes in low-income and middle-income countries remain undiagnosed and untreated (1). It is estimated that 46.5% of adults with diabetes were undiagnosed globally in 2015 (7). In the Chinese population, the prevalence of undiagnosed diabetes was 8.1%, with an estimated prevalence of 3.5% for those with previously diagnosed diabetes (2), implying that numerous Chinese adults with diabetes were undiagnosed.

According to American Diabetes Association (ADA) criteria, impaired fasting glucose (IFG) is defined as fasting plasma glucose (FPG) levels between 5.6 and 6.9 mmol/L and impaired glucose tolerance (IGT) is defined as 2-h plasma glucose values (2h-PG) after 75 g oral glucose tolerance test (OGTT) levels between 7.8 and 11.0 mmol/L. However, the World Health Organization (WHO) and numerous other diabetes organizations define the IFG cutoff at 6.1 mmol/L (8). Additionally, ADA recommends that it is reasonable to consider an A1c range of 5.7–6.4% as identifying individuals with prediabetes (8), while HbA1c is not considered to be a suitable diagnostic test for prediabetes by WHO and many other diabetes societies (9, 10). Screening for (pre)diabetes using FPG routinely but not OGTT is recommended in Chinese population by Chinese Diabetes Society, although the possibility of missed diagnosis was stated in the guideline (10). OGTT is well known as the gold standard diagnostic test in diabetes (11). Noteworthy, limited studies are available on the effects of not performing OGTT in detecting (pre)diabetes, including diagnosis efficiency, risk assessment of developing (pre)diabetes and probable costs, especially in Chinese Han population, which constitute the world’s largest ethnic group, making up around 20% of the global population (12), though it is reported recently that not performing an OGTT results in significant underdiagnoses of (pre)diabetes in a high-risk Caucasian population (13). In view that 47% of people with diabetes were aged between 40 and 59 years (14), and the prevalence peaked at ages 65–69 years for men and ages 75–79 years for women (7), we attempted to evaluate the performance mentioned above of OGTT vs FPG, 2h-PG and HbA1c in the screening of (pre)diabetes in Chinese Han population over 40 years in this study.

## Methods

### Study population and sampling

A total of 7200 eligible residents over 40 years in Hubei Province, located in central China, were selected and invited to participate in the study with a complex, multistage, probability sampling design. The overall response rate was 92.8% and 58 participants, whose data on FPG or 2h-PG in OGTT were missing, were excluded from analysis. When an individual was ineligible, refused or unavailable, a replacement household was substituted from the initial list, ensuring a sufficient sample size and representativeness of the data. Noteworthy, male individuals were not as willing as female to take a break to participate in the study since most of them were mainly self-employed, the only income-earner of their family, and preferred to work 7 days weekly. We were not able to refuse the families with some qualified members absence, otherwise a large number of sampled households would be excluded and replaced, which might introduce greater bias. The individuals detected as (pre)diabetes were referred to physician or specialties for further consultation or/and intervention. The follow-up survey was conducted 3 years later. All participants were advised to avoid mediations which might affect blood glucose levels before the visit if possible. The study was approved by the Ethics Committee of Tongji Medical College, Huazhong University of Science and Technology and was conducted in accordance with the Declaration of Helsinki. Written, informed consent was obtained from all the participants.

### Data collection and examination

Data collection was performed by the trained staff and a questionnaire was completed for gathering information on demographic characteristics and medical history. Participants without a self-reported history of diabetes were provided with a standard 75 g glucose solution, and blood sampling was conducted at 0 and 2 h after administration. Plasma glucose was measured using glucose oxidase method (15).

### Definitions

Persons are considered to have previously diagnosed (pre)diabetes if they respond ‘yes’ to the question ‘have you ever been told by a doctor, nurse or other health professional that you have (pre)diabetes.’ in the questionnaire. Diabetes mellitus was defined as FPG ≥7.0 mmol/L or 2h-PG ≥11.1 mmol/L (16, 17). Diabetes was divided into three subcategories: isolated fasting diabetes, isolated 2 h post-load diabetes and combined fasting and 2 h post-load diabetes. Prediabetic individuals were defined as IFG using ADA criteria (FPG levels 5.6–6.9 mmol/L) (16) and IFG using WHO criteria (FPG levels 6.1–6.9 mmol/L) (17) or IGT (2h-PG of 7.8–11.0 mmol/L) (16). Prediabetes included isolated IFG, isolated IGT and combined IFG and IGT (18). An A1C range of 5.7–6.4% was consider as identifying individuals with prediabetes and the criteria for diabetes diagnosis was ≥6.5% (16).

### Definition of costs

Costs were expressed in the equivalent of 2011–2012 Chinese Yuans (19). Health system costs were assessed based on the research data and practical price in China (19, 20), including costs of testing and costs for false-positive or false-negative results as described by Chatterjee *et al*. (21). Considering type 2 diabetes is the predominant form of diabetes in adults and type 1 diabetes is generally diagnosed soon since its symptoms usually develop very quickly (1), we attempted to perform the costs assessment mainly based on the evidence of type 2 diabetes. The references not distinguishing type 1 and type 2 diabetes were substituted when the data of type 2 diabetes is unavailable.

### Costs of testing

The direct medical costs of testing consisted of costs for laboratory tests, cost of OGTT glucose drink and staff costs (21, 22). The medical costs for FPG, 2h-PG, OGTT and HbA1c tests were 9.89, 23.56, 33.45 and 84.16, respectively. The nonmedical costs were calculated based on the report by Ye *et al*. (23). Nonmedical costs of testing, medical costs of testing and testing and follow-up testing costs in total and per true positive (TP) were calculated for cost analysis.

### Costs of false-positive or false-negative results

False-positive cases for prediabetes were the individuals supposed to be diagnosed with diabetes. False-negative cases were those supposed to be diagnosed with diabetes or prediabetes by OGTT but undetected by other testing strategies. Since early and better intervention can achieve concrete financial benefits in both the short and longer term, the cost of false-positive or false-negative cases included the cost increase from complications not avoided by lack of timely and appropriate intervention for diabetes. As no exact data are available in China, the cost increase was estimated with adjustment of discrepancy based on the cost reduction from complications avoided per person in the United Kingdom, which ranged from (GBP) £83 to £138, £317 to £622, £682 to £1366, £1078 to £1999 and £1280 to £2223 for type 2 diabetes patients over 5, 10, 15, 20 and 25 years, respectively (19, 24, 25).

### Statistical analyses

The data are presented as proportions (95% CI). The proportions of different glycemic status at baseline were evaluated by OGTT using WHO or ADA criteria. The proportions of detected or undetected diabetes were calculated based on medical history and the testing individually. Detecting strategies included FPG using WHO (FPG-WHO) or ADA (FPG-ADA) criteria, 2h-PG, OGTT using WHO (OGTT-WHO) or ADA (OGTT-ADA) criteria and HbA1c using ADA criteria. Sensitivity, specificity, false-negative rate and false-positive rate of different detecting strategies (FPG-WHO, FPG-ADA, 2h-PG and HbA1c) were calculated. Cross tabulation analysis was performed to investigate the association between glycemic status evaluated by different detecting strategies at baseline and by OGTT using respective criteria at follow-up. Data were analyzed with SPSS, version 13 (SPSS, Inc.).

## Results

### Prediabetes and diabetes prevalence and rate of diagnosis

The cohort demographics were as follows: 65.6% was female, mean age was 54.6 **± **8.4 years and 69.5% were from rural areas. The prevalence of isolated IFG, isolated IGT and combined IGT and IFG were estimated to be 11.1% (95% CI, 10.3–11.9), 13.9% (95% CI, 13.1–14.7) and 6.7% (95% CI, 6.1–7.3) using WHO criteria and 32.0% (95% CI, 30.8–33.1), 6.4% (95% CI, 5.9–7.1), 14.2% (95% CI, 13.3–15.0) using WHO criteria. The estimated prevalence of isolated fasting diabetes, isolated 2h post-load diabetes, combined fasting and post-load diabetes were 3.5% (95% CI, 3.1–4.0), 4.4% (95% CI, 3.9–4.9) and 3.6% (95% CI, 3.2–4.1). It was estimated that 6.9% (95% CI, 6.3–7.5) had been diagnosed previously among all individuals and 62.6% (95% CI, 59.8–65.3) had not been detected before among all individuals with diabetes. Notably, among individuals with diabetes, the proportion that had not been detected previously was 60.6% (95% CI, 56.2–65.2) in male and 63.7% (95% CI, 60.3–67.1) in female, 70.6% (95% CI, 67.4–73.9) in rural and 50.2% (95% CI, 46.0–55.2) in urbanized rural areas and ranged from 59.8% (95% CI, 52.6–66.0) to 63.9% (95% CI, 58.2–70.0) among individuals of different age (Table 1). Actually, we attempted to investigate the proportion of prediabetes diagnosed before in the participants, and few of them were aware of their condition about prediabetes. Thus, we were not able to provide the data in this investigation.

**Table 1:** Prediabetes and diabetes prevalence and rate of diagnosis.

	The proportion in total participants								The proportion in DM	
	Normal	Prediabetes			DM				Undetected DM	Detected DM
					Undiagnosed DM			Diagnosed DM		
		Isolated IFG	IFG and IGT	Isolated IGT	Isolated fasting DM	Fasting and PL DM	Isolated PL DM			
Overall										
WHO	3302 (49.8%)	735 (11.1%)	446 (6.7%)	919 (13.9%)	235 (3.5%)	239 (3.6%)	291 (4.4%)	457 (6.9%)	765 (62.6%)	457 (37.4%)
	48.6–51.1	10.3–11.9	6.1–7.3	13.1–14.7	3.1–4.0	3.2–4.1	3.9–4.9	6.3–7.5	59.8–65.3	34.7–40.2
ADA	1919 (29.0%)	2118 (32.0%)	939 (14.2%)	426 (6.4%)						
	27.9–30.1	30.8–33.1	13.3–15.0	5.9–7.1						
Sex										
Male										
WHO	1134 (49.7%)	295 (12.9%)	142 (6.2%)	277 (12.1%)	72 (3.2%)	67 (2.9%)	124 (5.4%)	171 (7.5%)	263 (60.6%)	171 (39.4%)
	47.5–51.7	11.5–14.3	5.2–7.3	10.7–13.5	2.5–3.9	2.2–3.7	4.5–6.4	6.4–8.7	56.2–65.2	34.8–43.8
ADA	651 (28.5%)	778 (34.1%)	279 (12.2%)	140 (6.1%)						
	26.7–30.5	32.1–36.1	10.9–13.5	5.1–7.1						
Female										
WHO	2168 (49.9%)	440 (10.1%)	304 (7.0%)	642 (14.8%)	163 (3.8%)	172 (4.0%)	167 (3.8%)	286 (6.6%)	502 (63.7%)	286 (36.3%)
	48.3–51.3	9.2–11.1	6.2–7.8	13.8–15.9	3.2–4.3	3.4–4.6	3.3–4.5	5.8–7.3	60.3–67.1	32.9–39.7
ADA	1268 (29.2%)	1340 (30.9%)	660 (15.2%)	286 (6.6%)						
	27.8–30.5	29.5–32.3	14.2–16.3	5.9–7.3						
Age, years										
40–47										
WHO	967 (61.3%)	148 (9.4%)	57 (3.6%)	196 (12.4%)	44 (2.8%)	41 (2.6%)	40 (2.5%)	84 (5.3%)	125 (59.8%)	84 (40.2%)
	58.8–63.7	7.9–10.9	2.8–4.6	10.8–14.1	2.0–3.7	1.8–3.4	1.8–3.3	4.2–6.5	52.6–66.0	34.0–47.4
ADA	622 (39.4%)	493 (31.3%)	157 (10.0%)	96 (6.1%)						
	37.0–41.9	29.0–33.4	8.5–11.5	5.0–7.3						
47–52										
WHO	611 (53.8%)	140 (12.3%)	60 (5.3%)	142 (12.5%)	34 (3.0%)	35 (3.1%)	44 (3.9%)	70 (6.2%)	113 (61.7%)	70 (38.3%)
	50.9–56.9	10.4–14.3	4.0–6.6	10.6–14.4	1.9–4.0	2.1–4.1	2.8–5.1	4.8–7.6	54.1–68.3	31.7–45.9
ADA	364 (32.0%)	387 (34.1%)	137 (12.1%)	65 (5.7%)						
	29.4–34.6	31.3–36.8	10.2–13.9	4.3–7.0						
52–57										
WHO	685 (48.3%)	164 (11.6%)	105 (7.4%)	200 (14.1%)	52 (3.7%)	51 (3.6%)	65 (4.6%)	95 (6.7%)	168 (63.9%)	95 (36.1%)
	45.6–51.0	9.9–13.3	6.1–8.8	12.4–16.0	2.8–4.7	2.7–4.7	3.5–5.7	5.4–8.0	58.2–70.0	30.0–41.8
ADA	385 (27.2%)	464 (32.7%)	213 (15.0%)	92 (6.5%)						
	24.9–29.4	30.3–35.1	13.3–16.8	5.3–7.8						
57–62										
WHO	541 (43.0%)	144 (11.5%)	106 (8.4%)	193 (15.4%)	47 (3.7%)	71 (5.6%)	55 (4.4%)	100 (8.0%)	173 (63.4%)	100 (36.6%)
	40.2–46.0	9.7–13.1	7.0–10.0	13.4–17.4	2.7–4.8	4.4–7.0	3.3–5.5	6.5–9.5	57.5–68.9	31.1–42.5
ADA	292 (23.2%)	393 (31.3%)	218 (17.3%)	81 (6.4%)						
	20.9–25.6	28.8–34.0	15.3–19.5	5.1–7.9						
62–75										
WHO	498 (40.3%)	139 (11.2%)	118 (9.5%)	188 (15.2%)	58 (4.7%)	41 (3.3%)	87 (7.0%)	108 (8.7%)	186 (63.3%)	108 (36.7%)
	37.3–43.1	9.4–13.0	7.9–11.2	13.3–17.2	3.6–5.9	2.3–4.4	5.7–8.5	7.3–10.4	57.8–68.4	31.6–42.2
ADA	256 (20.7%)	381 (30.8%)	214 (17.3%)	92 (7.4%)						
	18.4–22.9	28.3–33.6	15.0–19.5	6.0–8.9						
	The proportion in total participants								The proportion in DM	
	Normal	Prediabetes			DM				Undetected DM	Detected DM
					Undiagnosed DM			Diagnosed DM		
		Isolated IFG	IFG and IGT	Isolated IGT	Isolated fasting DM	Fasting and PL DM	Isolated PL DM			
Location										
Rural										
WHO	2333 (50.6%)	597 (13.0%)	336 (7.3%)	599 (13.0%)	192 (4.2%)	154 (3.3%)	178 (3.9%)	218 (4.7%)	524 (70.6%)	218 (29.4%)
	49.1–52.1	12.0–14.0	6.6–8.1	12.0–14.0	3.6–4.8	2.8–3.8	3.3–4.4	4.2–5.3	67.4–73.9	26.1–32.6
ADA	1284 (27.9%)	1646 (35.7%)	680 (14.8%)	255 (5.5%)						
	26.5–29.2	34.4–37.2	13.7–15.9	4.9–6.2						
Urbanized rural cluster										
WHO	969 (48.0%)	138 (6.8%)	110 (5.5%)	320 (15.9%)	43 (2.1%)	85 (4.2%)	113 (5.6%)	239 (11.8%)	241 (50.2%)	239 (49.8%)
	45.8–50.1	5.8–7.9	4.6–6.4	14.3–17.4	1.5–2.8	3.4–5.2	4.7–6.6	10.4–13.4	46.0–55.2	44.8–54.0
ADA	635 (31.5%)	472 (23.4%)	259 (12.8%)	171 (8.5%)						
	29.5–33.6	21.7–25.2	11.3–14.3	7.3–9.8						

Data are presented as the *n* (proportions) 95% CI.

2h-PG, 2-h plasma glucose; ADA, American Diabetes Association; CI, confidence intervals; DM, diabetes; FPG, fasting plasma glucose; IFG, impaired fasting glucose; IGT, impaired glucose tolerance; OGTT, oral glucose tolerance test; PL, 2h post-load; WHO, World Health Organization.

### Sensitivity, specificity and receiver-operating characteristic (ROC) analysis

**Figure 1:**
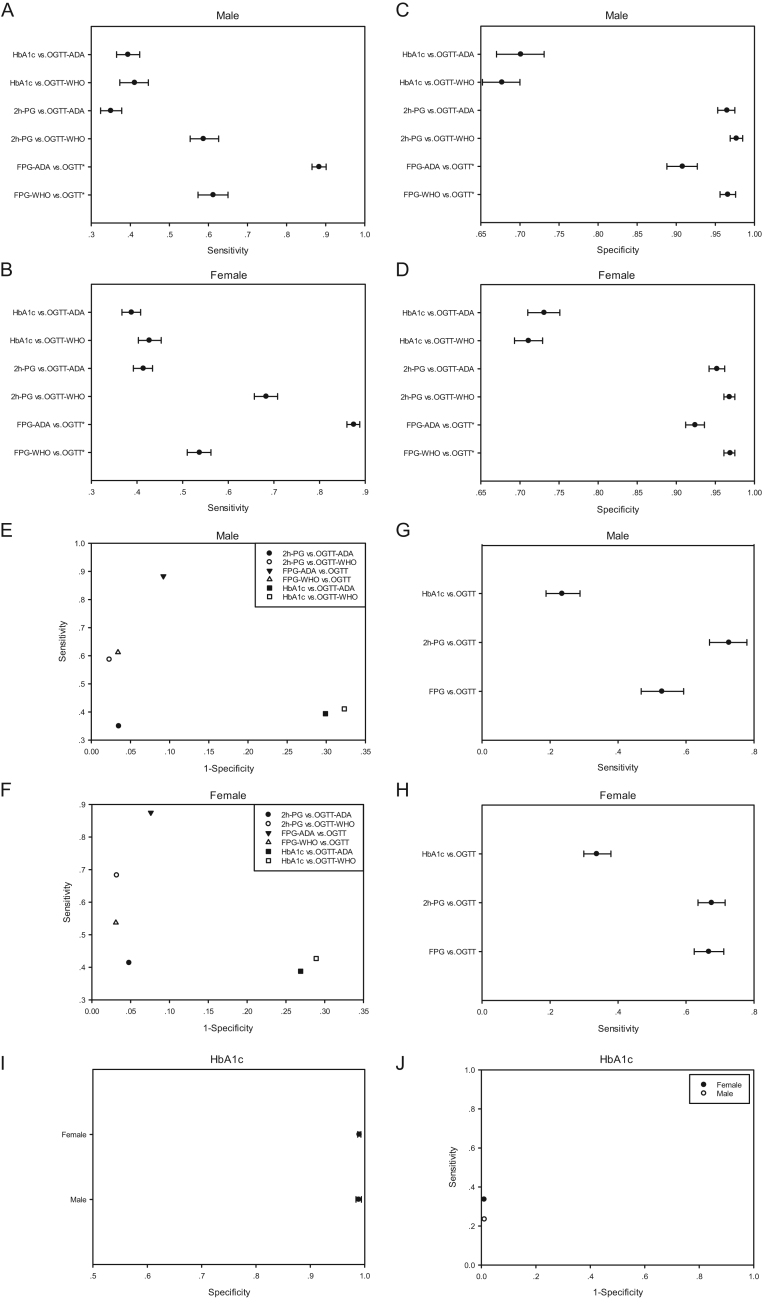
Sensitivity, specificity and receiver operating characteristic (ROC) analysis Data are presented as the n (proportions) 95% CI. 2h-PG, 2-h plasma glucose; ADA: American Diabetes Association; CI: Confidence intervals; FPG: fasting plasma glucose; OGTT: oral glucose tolerance test; WHO: World Health Organization; vs.: compared with OGTT using WHO or ADA criteria correspondingly.

In male, 61.2, 58.7 and 41.1% of individuals diagnosed with prediabetes using WHO criteria by OGTT, and 88.3, 35.0 and 39.4% using ADA criteria could be identified by FPG, 2h-PG and HbA1c testing separately (Fig. 1A), while they determined 53.7, 68.3, 42.7 and 87.5, 41.4 and 38.8% of individuals with prediabetes identified by OGTT using WHO or ADA criteria individually in female (Fig. 1B). For prediabetes determination, HbA1c testing had the lowest specificity both in male and female (Fig. 1C and D), while FPG testing using ADA criteria had the highest sensitivity and considerable specificity in male and female, and the ROC plots closely approached the top left hand corner of the graph (Fig. 1E and F).

Meanwhile, FPG, 2h-PG and HbA1c testing identified 52.9, 72.6 and 23.5% of individuals diagnosed with diabetes by OGTT in male (Fig. 1G) and 66.7, 67.5 and 33.7% in female (Fig. 1H), respectively. For diabetes identification, HbA1c testing had high specificity (Fig. 1I) but the ROC plots were far from the top left hand corner of the graph (Fig. 1J) in both male and female.

### Association of glycemic status evaluated by OGTT at follow-up and by different detecting strategies at baseline

**Figure 2:**
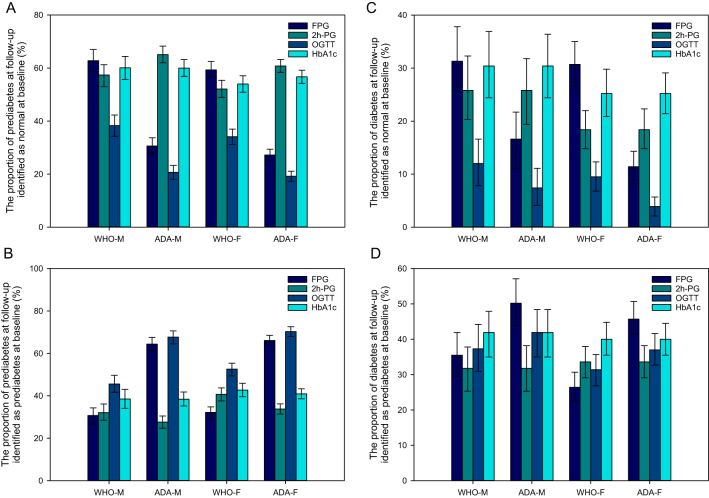
Association of glycemic status evaluated by OGTT at follow-up and by different diagnostic strategies at baseline Data are presented as the n (proportions) 95% CI. 2h-PG, 2-h plasma glucose; ADA: American Diabetes Association; CI: Confidence intervals; FPG: fasting plasma glucose; OGTT: oral glucose tolerance test; WHO: World Health Organization; vs.: compared with OGTT using WHO or ADA criteria correspondingly.

For the subjects diagnosed with prediabetes at follow-up, the lowest proportion developed from the normal glucose tolerant individuals (Fig. 2A) and the largest proportion derived from prediabetes (Fig. 2B) were both identified by OGTT using WHO or ADA criteria in male and female.

In the individuals determined to have diabetes at follow-up, the lowest proportion originated from normal glucose-tolerant subjects (Fig. 2C), and a large proportion from prediabetes group (Fig. 2D) were detected by OGTT using WHO or ADA criteria in both male and female, although larger proportions from those with prediabetes were detected by FPG testing using ADA criteria or by HbA1c.

### Costs estimation

**Figure 3:**
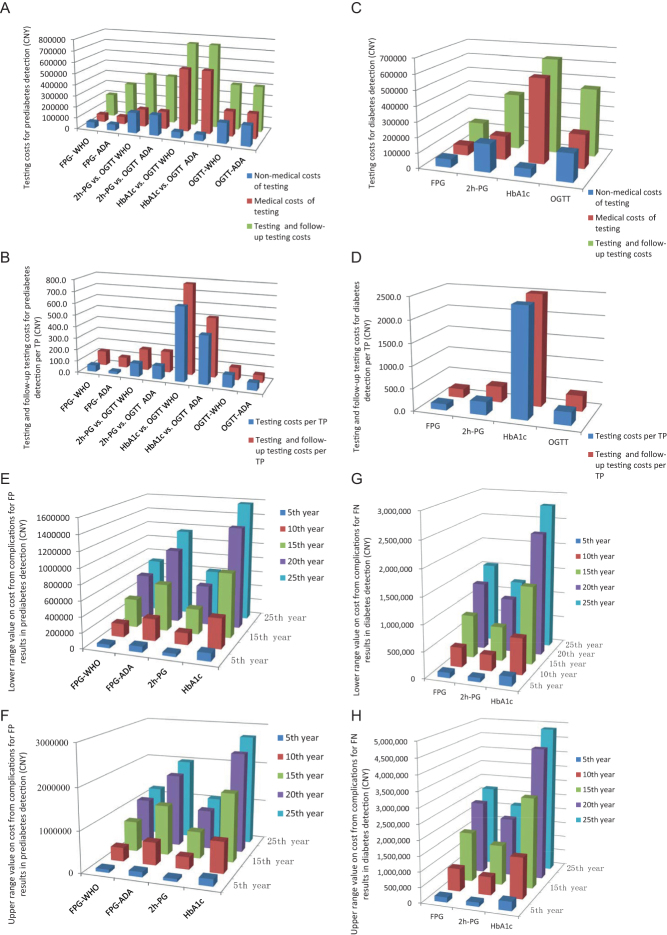
Costs estimation of different (pre)diabetes detecting strategies. (A) Testing costs for prediabetes. (B) Testing and follow-up testing costs for prediabetes detection per TP. (C) Testing costs for diabetes detection. (D) Testing and follow-up testing costs for diabetes detection per TP. (E) Lower range value on cost from complications for FP results in prediabetes detection. (F) Upper range value on cost from complications for FP results in prediabetes detection. (G) Lower range value on cost from complications for FN results in diabetes detection. (H) Upper range value on cost from complications for FN results in diabetes detection. TP, true positive case; FP, false positive; FN, false negative.

The total medical costs and nonmedical costs of testing were estimated to be 65511.4, 156061.4, 557475.8, 221572.8 and 54979.2, 182160, 54979.2, 182160 for FPG, 2h-PG, HbA1c and OGTT testing, respectively. The testing and follow-up testing costs were 200517.9, 320101.8, 428549.3, 740389.1 and 403732.8 for the prediabetes detecting strategies of FPG-WHO, FPG-ADA, 2h-PG, HbA1c and OGTT (Fig. 3A). The testing costs per TP were 55.5, 21.4, 114.3, 631.3, 412.0, 105.5 and 63.7 for the prediabetes-detecting strategies of FPG-WHO, FPG-ADA, 2h-PG, HbA1c-WHO, HbA1c-ADA, OGTT-WHO and OGTT-ADA, while their testing and follow-up testing costs per TP were 123.2, 86.7, 180.5, 776.2, 506.6, 105.5 and 63.7 (Fig. 3B). For diabetes, the testing and follow-up testing costs were 28890.3, 32303.5, 17370.75 and 46626.75 (Fig. 3C), the testing costs per TP case were 138.2, 294.5, 2423.8 and 289.6 and the testing and follow-up testing costs per TP case were 199.2, 355.4, 2499.3 and 350.6 for detecting strategies of FPG, 2h-PG, HbA1c and OGTT (Fig. 3D).

The estimated cost from complications not avoided by lack of timely and proper intervention per person in China estimated with adjustment of discrepancy in the medical cost ranged from (CNY) 341 to 567, 1302 to 2555, 2802 to 5611, 4428 to 8212 and 5258 to 9132 for people with type 2 diabetes over 5, 10, 15, 20 and 25 years, respectively (19, 24, 25). The false-positive individuals for prediabetes detecting were 132, 218, 117 and 229 using FPG-WHO, FPG-ADA, 2h-PG and HbA1c strategies. Overall, 229 of the individuals diagnosed with prediabetes by HbA1c were supposed to be diagnosed with diabetes, while 917 and 447 of them had normal glucose tolerance using WHO or ADA criteria, respectively. Noteworthy, 291, 235 and 531 individuals had false-negative results for prediabetes on detection using FPG, 2h-PG and HbA1c strategies, respectively. The estimated cost from complications unavoided per person due to the lack of proper intervention for false-positive results of FPG, 2h-PG and HbA1c strategies ranged from 45006 to 1990745, 39892 to 1068427 and 101946 to 2730426 in prediabetes detection (Fig. 3E and F) and for false-negative results from 99218 to 2657371, 80125 to 2145987 and 181047 to 4849017 in diabetes detection (Fig. 3G and H) per year in the 5th–25th year.

## Discussion

In this study, we demonstrated that the prevalence of isolated IGT and isolated 2h post-load diabetes were high, especially using WHO criteria, and more than 60% of individuals with diabetes were undetected. Compared with OGTT, FPG testing identified around 60% prediabetes using WHO criteria and more than 80% using ADA criteria, which were much higher than 2h-PG and HbA1c testing, while 2h-PG testing determined around 70% individuals with diabetes, which was superior to FPG and HbA1c. Additionally, the individuals least identified as normal glucose tolerant at baseline by OGTT would develop prediabetes or diabetes after 3 years. The highest proportion of prediabetes individuals and a large proportion of diabetes patients at follow-up originated from the prediabetes at baseline group identified by OGTT, although the highest proportion of diabetes individuals were from prediabetes group diagnosed by FPG using ADA criteria or HbA1c. Moreover, FPG testing and OGTT were the least expensive strategies for (pre)diabetes detection, with HbA1c testing is the most expensive. Notably, FPG, 2h-PG and HbA1c strategies, not performing OGTT, would increase cost from complications for false-positive (FP) or false-negative (FN) results, with 2h-PG being the least expensive and HbA1c being the most expensive.

In this investigation, the prevalence of isolated IGT was higher than that of isolated IFG, or combined IGT and IFG using WHO criteria. Likewise, the prevalence was much lower using ADA criteria but still as high as 6.4%. Additionally, the estimated prevalence of isolated 2h post-load diabetes was higher than isolated fasting diabetes or combined fasting and post-load diabetes. These data implied that not performing OGTT would result in significant underdiagnosis of (pre)diabetes in Chinese Han population over 40 years, which are consistent with the findings obtained in overweight and obese adult Caucasian population (13). Moreover, these findings suggested that detecting dysglycemia using WHO diagnostic criteria without performing OGTT would have more risk of prediabetes underdiagnosis in the selected population, which are being widely adopted in China (10), while using ADA criteria was helpful to decrease the risk of prediabetes underdiagnosis in these individuals if OGTT was not available. Moreover, our data showed that the majority of individuals with diabetes were undetected, in male and female, in all age ranges over 40 years old, in rural and urbanized rural areas. The overall prevalence of undiagnosed diabetes was around 11.5% and the prevalence of previously diagnosed diabetes was 6.9%, which were higher than the findings obtained in large and nationally representative samples in Chinese adults aged 18 years or older by Xu *et al*. (2) or aged 20 years or older by Yang *et al*. (18). Recently, a geographical variation analysis in diabetes prevalence and detection in China by a nationally representative health survey of adults aged ≥18 years illustrates that the diabetes prevalence is 9.3–11.5% (26). The higher diabetes prevalence in this study may be attributed to the prevalence increasing with age (7). Thus, more attention would be necessary to be paid to (pre)diabetes detection in individuals aged ≥40 years.

Noteworthy, our data showed that FPG-ADA had the highest sensitivity in detecting prediabetes, with close and high-ranked specificity to FPG-WHO, 2h-PG-WHO and 2h-PG-ADA. It is reported that FPG cutoff points lower than 6.1 mmol/L are helpful to screen prediabetes (22), which is consistent with our observations. Actually, FPG-WHO strategy has been being widely employed in China for prediabetes detection (27). Thus, there should be rational consideration of the future role of FPG testing strategy using ADA criteria in detecting prediabetes in Chinese Han population if OGTT is not available. Additionally, 2h-PG testing had good sensitivity and specificity in prediabetes detection and its sensitivity seemed better than FPG or HbA1c strategies for diabetes detection in this study, which is also consistent with the data that not performing an OGTT results in significant underdiagnosis of (pre)diabetes in adult Caucasian population (13). Therefore, strong consideration should be given to OGTT in (pre)diabetes detection in Chinese Han population. Notably, the sensitivity and specificity of HbA1c testing were not so good as FPG or 2h-PG-WHO strategies in this investigation, which are consistent with the findings in Americans (28). Recently, this inadequacy of HbA1c as a screening tool for prediabetes is also reported in Japanese population (29). Herein, there should be careful thinking of the future role of HbA1c testing strategy in detecting (pre)diabetes in Chinese Han population.

Our data also demonstrated that for (pre)diabetes detection, FPG testing was the least expensive in testing and follow-up testing costs in total or compared per TP, with HbA1c testing being the most expensive. Interestingly, when costs were compared per TP identified, FPG-ADA strategy was the most cost-saving and FPG-WHO was the secondary, while their costs were close to OGTT-ADA. However, our results indicated that FPG, 2h-PG and HbA1c would increase cost from complications for FP or FN results, with 2h-PG testing strategy being the least expensive and HbA1c being the most expensive. These results are consistent with the findings obtained in the United States by Chatterjee *et al*., suggesting that the use of glucose challenge test approach was the least expensive test from perspectives when costs were compared per TP identified (21). These cost analysis data also supported strong consideration should be given to OGTT in (pre)diabetes detection in Chinese Han population. Furthermore, we found that the least individuals identified as normal glucose tolerant by OGTT at baseline would develop (pre)diabetes after 3 years, which also illustrates the superiority of OGTT in detecting (pre)diabetes (30). Importantly, the highest proportion of individuals identified as prediabetes at baseline by HbA1c or FPG testing using ADA criteria respectively developed diabetes after 3 years, which is interestingly and coincidentally consistent with the findings that HbA1c can identify more cardiovascular and metabolic risk profile in OGTT-negative Chinese population (31). These data implied the pivotal role of HbA1c or FPG testing using ADA criteria in predicting the risk of developing diabetes for individuals with prediabetes (32).

It should be noted that we were not able to determine the costs of different detection strategies in a cohort prospective study, although we tried to analyze these costs using excellent ideas and methods reported previously (20, 21, 22, 23) with adjustment of discrepancy based on the economic and medical status of China (25, 33). Longer follow-up on complications and costs are necessary to confirm the hypothesis put forward by this investigation. We did not show the information of micro- and macro-vascular complications in view that 3-year follow-up was probably not long enough to evaluate the complications comprehensively, though hyperglycemia has been widely considered to be closely related with vascular complications (34). Moreover, we performed the comparison between FPG, 2h-PG or HbA1c and OGTT, in view that OGTT is widely considered as gold standard diagnostic test in prediabetes and type 2 diabetes (11, 35). However, this standpoint is otherwise negated in the recent years (36). Herein, longer follow-up on the risk and probable costs of complications based on different testing strategies are needed to confirm the hypothesis put forward in this investigation.

### Conclusions

Notwithstanding these limitations, we found that the prevalence of isolated IGT and isolated 2h post-load diabetes were high and the majority of individuals with (pre)diabetes were undetected in Chinese Han population. We demonstrated that in a large population not performing an OGTT results in underdiagnosis, inadequate developing risk assessment and probable cost increases of (pre)diabetes in Han Chinese over 40 years. Thus, we tentatively put forward that great consideration should be given to OGTT in detecting (pre)diabetes in this population. Further research of population-based prospective cohort of longer-term effects is necessary to investigate the risk assessment and cost of (pre)diabetes.

## Declaration of interest

The authors declare that there is no conflict of interest that could be perceived as prejudicing the impartiality of the research reported.

## Funding

This work was supported by grants from the Ministry of Science and Technology of People’s Republic of China (2016YFbib901200 and 2016YFbib901203), National Natural Science Foundation of China (81471069, 81770843, 81800762 and 71601077), Natural Science Foundation of Hubei Province (2017CFB257) and Union Hospital, Tongji Medical College, Huazhong University of Science and Technology (02.03.2017-328 and 02.03.2017-332).
